# A Case of Recurrent Bacteremic Cellulitis Caused by Citrobacter koseri

**DOI:** 10.7759/cureus.89800

**Published:** 2025-08-11

**Authors:** Yuto Naito, Yoshiki Kusama, Hiroaki Terada

**Affiliations:** 1 General Internal Medicine, Saiseikai Senri Hospital, Suita, JPN; 2 Infectious Diseases, Osaka University Hospital, Suita, JPN

**Keywords:** antimicrobial prophylaxis, citrobacter, gram-negative bacteremia, lower-extremity lymphedema, refractory cellulitis, sepsis in liver cirrhosis

## Abstract

We encountered a rare case of recurrent *Citrobacter koseri* cellulitis with bacteremia in an 82-year-old woman with a history of surgery for cervical cancer and chronic lower limb lymphedema. She was admitted with cellulitis in the right lower leg and subsequently developed septic shock. Blood cultures revealed *C. koseri*, which was treated successfully with meropenem. Eight months later, she developed septic shock and cellulitis while hospitalized for lumbar compression fractures. Again, blood cultures yielded *C. koseri*. She recovered after treatment with cefmetazole but experienced a third episode of *C. koseri* cellulitis with bacteremia, which was treated with cefazolin. Long-term prophylactic cefalexin was initiated because of the frequent relapses. Although dementia-related nonadherence led to three further cellulitis episodes without bacteremia, a switch to once-daily levofloxacin improved adherence and prevented further recurrences. *Citrobacter koseri* is an uncommon cause of cellulitis and typically occurs in immunocompromised individuals. Risk factors in this case included lymphedema, cirrhosis, and multiple comorbidities. The formation of biofilm may have contributed to the recurrences. This case highlights the limitations of penicillin-based prophylaxis for Gram-negative cellulitis and suggests cefalexin as an alternative in selected cases. With population aging, Gram-negative soft tissue infections may become more common, increasing the need for preventive strategies tailored to resistant pathogens.

## Introduction

*Citrobacter* species are Gram-negative bacilli belonging to the Enterobacteriaceae family. They are widely distributed in nature and are commonly found in water, soil, and the intestinal tracts of humans and animals. Clinically, they are known to cause opportunistic infections, including urinary tract infections and catheter-related bloodstream infections, especially in hospitalized patients [1]. There are 11 species of *Citrobacter*. According to a 2024 systematic review of hospitalized patients in 28 observational or surveillance studies, *Citrobacter freundii* was the most frequently reported (79%), followed by *Citrobacter koseri* (39%) and *Citrobacter braakii* (18%) [2]. *Citrobacter koseri* has been reported to occasionally cause meningitis and brain abscesses in neonates and immunocompromised individuals. Despite its rarity, these infections have high morbidity and mortality; according to a comprehensive review, approximately one-third of infants with CNS abscesses die, and about one-half of survivors sustain neurologic sequelae [3].

Purulent cellulitis is an acute bacterial infection of the dermis and subcutaneous tissue and is typically characterized by spreading erythema, warmth, tenderness, and swelling. Risk factors for recurrent cellulitis include chronic edema, dermatophytosis, venous insufficiency, and obesity [4]. The most common causative organisms are Gram-positive cocci, particularly β-hemolytic streptococci, whereas Gram-negative bacilli are rare. In a review of erysipelas and cellulitis with bacteremia, blood cultures were positive in 4.6% of patients with erysipelas and 7.9% of those with cellulitis, with Gram-negative bacilli, respectively, accounting for 11% and 28% of these cases [5]. Among the Gram-negative bacilli, a *Citrobacter* species was identified in only one case of erysipelas and in none with cellulitis.

This report describes a rare case of recurrent episodes of cellulitis and bacteremia caused by *C. koseri* in an elderly woman with chronic lymphedema and reviews the literature on soft tissue infections caused by this organism.

## Case presentation

An 82-year-old woman presented to the emergency department with complaints of fever and lower leg swelling. She had been hospitalized one week earlier for cellulitis of unknown origin, during which her symptoms had improved. However, immediately after discharge, her lower leg edema recurred, and she developed fever, prompting readmission for further evaluation and treatment. Her medical history included cervical cancer treated by radical hysterectomy with pelvic lymphadenectomy and lumbar spinal canal stenosis. She was a resident of a nursing care facility.

**Figure 1:**
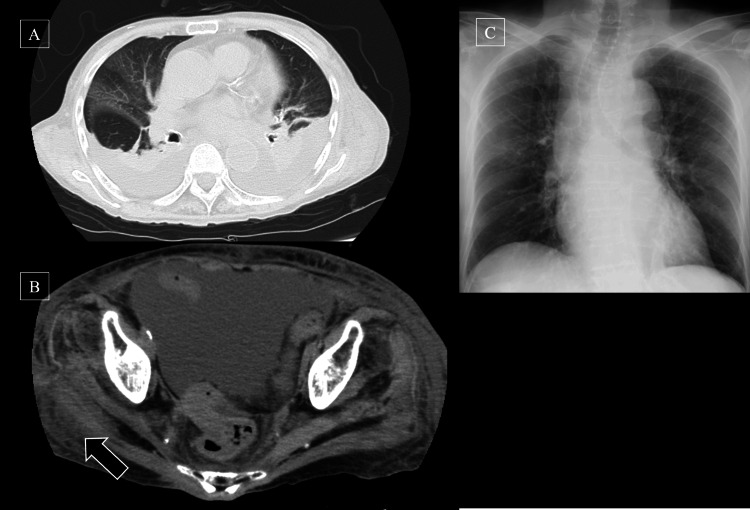
Radiological findings (A) Chest findings on plain computed tomography at the first admission: no apparent abnormalities were noted, except for ascites likely due to cirrhosis. (B) Leg findings on plain computed tomography at the first admission: swelling and fat stranding were observed in the right leg (arrow). (C) Chest X-ray at the second admission: no apparent abnormalities in the lungs or heart

On presentation, she had a heart rate of 113 beats per minute (bpm), a blood pressure (BP) of 122/66 mmHg, and a temperature of 39.7°C. Lung sounds were clear without rales, and heart sounds were normal. Bilateral lower leg edema was present, with tension, redness, and warmth in the right lower leg. Laboratory tests showed mild elevation of C-reactive protein (Table 1). Computed tomography (CT) scan revealed swelling and fat stranding in the right leg. It also detected bilateral pleural effusion, which was considered to be due to cirrhosis, and did not significantly impact the current symptoms (Figure 1A, 1B).

**Table 1:** Laboratory findings on initial admission ALT, alanine aminotransferase; AST, aspartate aminotransferase; CRP, C-reactive protein; RBC, red blood cells; WBC, white blood cells; BUN, blood urea nitrogen

Parameter	Value	Unit	Reference Value
WBC	4.5	10^9^/L	3.4-8.9
Neutrophil	65.9	%	35.9-69.8
RBC	3.14	10^12^/L	3.75-5.12
Hemoglobin	9.9	g/dL	10.2-14.9
Platelet count	128	10^9^/L	163-412
Total bilirubin	0.3	mg/dL	0.2-1.2
AST	24	IU/L	8-37
ALT	12	IU/L	0-39
BUN	16	mg/dL	9-20
Creatinine	1	mg/dL	0.4-0.7
CRP	0.23	mg/dL	≤0.18

First admission

The patient was diagnosed with cellulitis in the right lower leg based on physical examination and CT findings. Blood cultures were obtained, and she was started on intravenous cefazolin 2 g every eight hours. On hospital day 2, she developed shock with hypotension (systolic BP: 70 mmHg) that was unresponsive to fluids. A central venous catheter was inserted, and norepinephrine was started at 0.05 mg/kg/hour. On day 3, two sets of blood cultures obtained at admission yielded *C. koseri* as a pure culture, confirming septic shock secondary to cellulitis. Cefazolin was switched to meropenem 1 g every 12 hours. Her BP improved thereafter. Chest and abdominal computed tomography on day 4 showed pleural effusion and ascites. Diuretic therapy consisting of furosemide 20 mg/day, spironolactone 50 mg/day in two divided doses, and tolvaptan 7.5 mg/day was started. Norepinephrine was tapered off by day 9, and the central line was removed on day 11. Meropenem was discontinued on day 16. The patient improved and was transferred to a rehabilitation facility on day 22.

Second admission

Eight months later, the patient fell from a chair and was unable to walk. She was taken to a local clinic, where lumbar compression fractures were suspected, and she was referred to our hospital. X-rays showed compression fractures at L1 and L2, and she was admitted to the orthopedic department for pain management, immobilization using a corset, and rehabilitation.

On day 8, she developed chills and a high-grade fever with hypoxia (SpO₂: <90%) and hypotension (systolic BP: 60 mmHg). Erythema and warmth were observed from the lower abdomen to the lower legs. Chest X-ray shows no apparent abnormalities in the lungs or heart (Figure 1C). She was diagnosed with septic shock secondary to cellulitis and treated with hydration, meropenem 500 mg every 12 hours, and norepinephrine 0.05 mg/kg/hour. Norepinephrine was discontinued by day 10. On day 12, blood cultures from day 8 revealed *C. koseri*, and her antibiotic therapy was changed to cefmetazole 1 g every eight hours based on susceptibility results (Table 2). On day 15, follow-up cultures were negative, and oral levofloxacin was initiated on day 20. Rehabilitation was resumed thereafter.

**Table 2:** Antimicrobial susceptibility of Citrobacter koseri isolates during each episode S/A, sulbactam and ampicillin; T/P, tazobactam and piperacillin; S/T, sulfamethoxazole and trimethoprim; MIC, minimum inhibitory concentration

	First Episode		Second Episode		Third Episode	
	MIC	Interpretation	MIC	Interpretation	MIC	Interpretation
Ampicillin	>16	Resistance	>16	Resistance	>16	Resistance
Piperacillin	64	Intermediate	64	Intermediate	>64	Resistance
Cefazolin	≤2	Susceptible	≤2	Susceptible	≤2	Susceptible
Cefotiam	≤8	Susceptible	≤8	Susceptible	≤8	Susceptible
Ceftriaxone	≤1	Susceptible	≤1	Susceptible	≤1	Susceptible
Cefmetazole	≤16	Susceptible	≤16	Susceptible	≤16	Susceptible
Meropenem	≤1	Susceptible	≤1	Susceptible	≤1	Susceptible
S/A	≤8	Susceptible	≤8	Susceptible	≤8	Susceptible
T/P	≤16	Susceptible	≤16	Susceptible	≤16	Susceptible
Minocycline	≤4	Susceptible	≤4	Susceptible	≤4	Susceptible
Levofloxacin	≤0.12	Susceptible	≤0.12	Susceptible	≤0.12	Susceptible
S/T	≤2	Susceptible	≤2	Susceptible	≤2	Susceptible

However, on day 41, the patient again developed fever, erythema, and warmth in the lower limbs. Recurrent cellulitis and bacteremia were suspected, and cefazolin was started. Blood cultures taken on day 41 yielded *C. koseri* by day 44. The clinical course improved, and cefazolin was switched to oral cephalexin 1000 mg/day in four divided doses on day 68. Long-term prophylactic cefalexin was planned in view of the repeated episodes of cellulitis. She was discharged on day 95.

Post-discharge course

**Figure 2:**
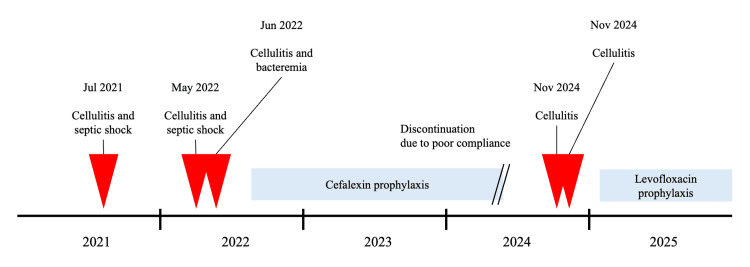
Overall clinical course in the present case Recurrent episodes of cellulitis were observed after the discontinuation of prophylactic oral cefalexin. The recurrences ceased following the initiation of prophylactic oral levofloxacin

There were no further recurrences of cellulitis following the initiation of prophylactic cefalexin. However, the patient developed worsening dementia, which negatively impacted her medication adherence. She had three episodes of cellulitis without bacteremia over the subsequent two-year period. To improve adherence, the cefalexin was replaced with once-daily levofloxacin 500 mg. No recurrence was noted in the four months following the switch. The patient’s overall clinical course is summarized in Figure 2.

## Discussion

This patient had a history of cervical cancer surgery and chronic lymphedema in the right lower limb, likely due to lymph node dissection. She experienced three episodes of *C. koseri* cellulitis with bacteremia and three additional episodes of cellulitis without bacteremia over a two-year period. Oral cefalexin and levofloxacin suppressed any further recurrences.

Peralta et al. investigated patients with cellulitis according to whether or not they developed bacteremia and found that having two or more comorbidities and edema were significant risk factors for bacteremia [6]. Our patient had lower leg edema following lymph node dissection, which may have increased the likelihood of cellulitis being accompanied by bacteremia. In general, Gram-positive bacteria are the predominant pathogens in cellulitis, whereas Gram-negative bacteria are rare [5,6]. However, patients with liver cirrhosis are more prone to Gram-negative cellulitis. A study in South India reported that 73% of cases of cellulitis in patients with cirrhosis were caused by Gram-negative bacteria [7]. Our patient had Child-Pugh class B cirrhosis, a condition conducive to Gram-negative infections.

Soft tissue infections in which the causative organism is *C. koseri* are extremely rare. A literature review identified only seven reported cases (Table 3) [8-14]. Three of these cases involved young immunocompetent individuals with acne, and two occurred in patients with hematologic malignancy. While the association between *C. koseri* and acne is interesting, it was not relevant in this case.

**Table 3:** Reported cases of skin and soft tissue infections caused by Citrobacter koseri Sources: [8-14] NR: not reported

Year	Authors	Age	Sex	Site	Presentation	Underlying Disease
1998	Bishara et al.	NR	NR	NR	Cellulitis	Multiple myeloma
2000	Chastain	28	Male	Face	Folliculitis	Acne vulgaris
2002	Garcia-Bustinduy et al.	49	Male	Scalp	Folliculitis	Alopecia
2010	Kluger et al.	60	Female	Lower limb	Cellulitis	Chronic B-cell lymphocytic leukemia
2015	Raia et al.	15	Male	Face	Folliculitis	Acne vulgaris
2021	Licata et al.	80	Female	Lower limb	Bullous erysipelas	Diabetes mellitus
2025	Khreis et al.	16	Female	Axilla	Abscess and cellulitis	Acne vulgaris

*Citrobacter* koseri can form biofilm through initial adhesion to tissue surfaces, followed by the secretion of extracellular polymeric substances. Biofilm formation has been reported in 10 of 11 isolates of *C. koseri* cultured from contact lenses [15]. In chronic lymphedema, protein-rich interstitial fluid, impaired lymphatic drainage, and reduced antibiotic penetration may facilitate this process. Biofilm development can begin within hours after adhesion and mature within several days, potentially contributing to persistent and recurrent infection in our patient.

Phenoxymethylpenicillin is commonly used as prophylaxis against recurrent cellulitis and has been shown to reduce the risk of recurrence by 45% [16]. However, *C. koseri* is intrinsically resistant to penicillins, including amoxicillin. Therefore, we used cephalexin for prophylaxis. Although there are few reports on the prophylactic use of cephalexin for recurrent cellulitis, it was effective until the patient’s compliance deteriorated. Cephalexin may be a viable option for prophylaxis against penicillin-resistant pathogens; however, caution is warranted in view of the risk of resistance. Cotrimoxazole is also active against *C. koseri* and could be considered for prophylaxis; however, in this case, levofloxacin was chosen because its once-daily dosing was expected to provide better compliance in a patient with dementia. In this case, we planned long-term prophylactic therapy for a minimum duration of 12 months because of the patient’s persistent predisposing factors, such as chronic lymphedema and cirrhosis. Discontinuation was to be considered if no recurrence occurred during this period, whereas recurrence after discontinuation would prompt the resumption of prophylaxis. This approach was chosen to balance recurrence prevention with concerns about antimicrobial resistance. An increase in Gram-negative soft tissue infections has recently been reported, likely owing to aging populations, and Japan’s aging society may face similar trends [17]. Although *C. koseri* in this case remained susceptible to non-penicillin antibiotics, Gram-negative bacteria generally show high resistance to these drugs. Broad-spectrum antibiotics are often required for treatment and prevention, which may contribute further to the development of resistance. Guidelines for the prevention of recurrent cellulitis caused by Gram-negative bacteria may become necessary in the future, but more research is needed to establish effective prevention strategies.

## Conclusions

We report a rare case of recurrent *Citrobacter koseri* cellulitis with bacteremia in an elderly woman with chronic lymphedema and multiple comorbidities, including liver cirrhosis. Despite appropriate antibiotic treatment, she experienced repeated recurrences. Long-term prophylaxis with cephalexin, and later levofloxacin, proved effective in preventing further episodes. This case underscores the importance of considering Gram-negative organisms in recurrent cellulitis, especially in elderly or immunocompromised patients. With the aging population and increasing antimicrobial resistance, clinicians may need to broaden their preventive strategies and antibiotic choices to include coverage for Gram-negative pathogens.
